# MysiRNA-Designer: A Workflow for Efficient siRNA Design

**DOI:** 10.1371/journal.pone.0025642

**Published:** 2011-10-26

**Authors:** Mohamed Mysara, Jonathan M. Garibaldi, Mahmoud ElHefnawi

**Affiliations:** 1 Informatics and Systems Department and Biomedical Informatics and Chemoinformatics Group, Division of Engineering Research and Centre of Excellence for Advanced Sciences, National Research Centre, Cairo, Egypt; 2 Information Technology Institute, Division of Biomedical Informatics and Bioinformatics, Smart Village, 6th of October City, Egypt; 3 Computer Science and Engineering Department, Egypt-Japan University of Science and Technology (E-JUST), Alexandria, Egypt; 4 Intelligent Modeling and Analysis Research Group, School of Computer Science, University of Nottingham, Nottingham, United Kingdom; Niels Bohr Institute, Denmark

## Abstract

The design of small interfering RNA (siRNA) is a multi factorial problem that has gained the attention of many researchers in the area of therapeutic and functional genomics. *MysiRNA* score was previously introduced that improves the correlation of siRNA activity prediction considering state of the art algorithms. In this paper, a new program, *MysiRNA-Designer*, is described which integrates several factors in an automated work-flow considering mRNA transcripts variations, siRNA and mRNA target accessibility, and both near-perfect and partial off-target matches. It also features the *MysiRNA* score, a highly ranked correlated siRNA efficacy prediction score for ranking the designed siRNAs, in addition to top scoring models Biopredsi, DISR, Thermocomposition21 and i-Score, and integrates them in a unique siRNA score-filtration technique. This multi-score filtration layer filters siRNA that passes the 90% thresholds calculated from experimental dataset features. *MysiRNA-Designer* takes an accession, finds conserved regions among its transcript space, finds accessible regions within the mRNA, designs all possible siRNAs for these regions, filters them based on multi-scores thresholds, and then performs SNP and off-target filtration. These strict selection criteria were tested against human genes in which at least one active siRNA was designed from 95.7% of total genes. In addition, when tested against an experimental dataset, *MysiRNA-Designer* was found capable of rejecting 98% of the false positive siRNAs, showing superiority over three state of the art siRNA design programs. *MysiRNA* is a freely accessible (Microsoft Windows based) desktop application that can be used to design siRNA with a high accuracy and specificity. We believe that *MysiRNA-Designer* has the potential to play an important role in this area.

## Introduction

siRNAs are small double-stranded non-coding RNA molecules capable of utilizing the RNA interference gene regulatory mechanism. As such, they are capable of down-regulating mRNA and causing targeted gene silencing. This induced gene silencing is naturally utilized to target foreign genetic elements inside cells and has been utilized extensively to identify gene functions (functional genomics studies) or even (as an ultimate goal) treat certain gene-mediated diseases such as Cancer. For this reason, siRNAs have become a core interest of many biological research laboratories in the last decade. Several efforts have been made to rationalize siRNA design, starting with Tuschl principles [1], Reynolds [2], Amarzguioui [3], Takasaki [4], Katoh [5], Ui-Tei [6], and Hsieh [7] who developed some of the first-generation position dependant tools for siRNA design that had a relatively low correlation to actual siRNA activity [8].

This was followed by second-generation tools such as Biopredsi [9], ThermoComposition21 [10], DSIR [11], i-Score [8], siRNA Scales [12], using intelligent data-mining approaches. Although these tools provide guidance for evaluating the siRNA-mRNA binding, and predicting their silencing efficiency, other aspects need to be taken into consideration for proper design of siRNAs with high specificity and sensitivity. The first aspect is alternative splicing, as the entire gene transcripts should be assigned for targeting and only the conserved regions between multiple transcripts should be targeted, as one mismatch between alternative transcripts and siRNA may dramatically affect siRNA efficiency [13], [14]. In the experiment carried out by Czaudema, there was noticeable decrease in the efficacy of designed siRNA when central single nucleotide variation was induced between the siRNA and the targeted mRNA [15].

The second aspect is target accessibility and thermodynamic features of both siRNAs and targeted mRNAs, for which several studies have been performed to investigate thermodynamic features affecting siRNA functionality. These features include thermodynamic differential end instability as a key feature reported in different studies [16], [17], unstructured guide strands (unstable siRNA secondary structure) [18], and high probability of siRNA terminal-ends to single-stranded (unpaired) nucleotides [19], [20]. All of these affect siRNA and mRNA binding, and are correlated with their silencing efficiency [18], [21]. Target mRNA accessibility evaluation is crucial for proper designing of efficient siRNA, as mRNA tends to form secondary structure that affects its accessibility and hence reduces the capability to design siRNA targeting certain regions of mRNA. Therefore, target accessibility evaluation represents an important cornerstone and rate-limiting step in siRNA design and selection. The effect of target secondary structure and RNA interference was extensively studied using different datasets ranging from 100 siRNA targeting three genes, to 3,084 siRNAs targeting 82 genes, showing correlation between secondary structure and interference efficiency [22]–[26]. It has been suggested that siRNA structure affects it efficiency by reducing its ability to bind to the target site and/or hindering RISC-siRNA interaction [18].

Several siRNA sequence features affect structural accessibility, such as GC-rich regions and palindrome regions that lead to the formation of stable intra-molecular structures [27]. Moreover, the energetic calculations are considered another aspect to evaluate siRNA-mRNA target accessibility. Since the interaction between two RNA sequences (siRNA and mRNA) requires energy in two distinctive phases: the phase where energy is needed to open the binding site (mRNA opening energy) and the opening of the siRNA duplex (siRNA guide-strand release), and the second phase where energy is required for the hybridization between the guide-strand and the mRNA. The summation of these two energies is defined as the total interaction energy. The energy required for opening the siRNA duplex and mRNA should be less than the hybridization energy between siRNA and the mRNA. There is evidence of the correlation between siRNA inhibition efficiency and siRNA-mRNA binding energy [28] that strengthens the findings of Ladunga, in which target accessibility information was found to provide the most predictive feature among the 142 features studied and improve the prediction of highly efficient siRNA [21]. Upon testing siRNAs against gradually less accessible target sites, it showed that there was correlation between the target accessibility and the siRNA efficiency [28], [29].

The third aspect is off-target filtration, as single siRNA could be targeting several mRNA targets by either sense or antisense [30]. “Ideally, the siRNA must not cause any effects other than those related to the knock down of the target gene” [31]. Two main mechanisms have been identified for siRNA being induced off-target: either by provoking innate immunity effects or by complete/partial homology with unintended mRNA [27]. The innate immunity effect is caused either by cytosolic double-stranded RNA (dsRNA) immunorecognition that could be avoided by using siRNA with length less than 30 nts [32], or triggered via Toll-like receptor 7 sequence-dependent immunorecognition. Although siRNAs with length less than 30 nts avoid Cytosolic dsRNA immunorecognistion they are capable of triggering Toll-like receptor 7 recognition[18]. Identification of motifs such as 5′-GUCCUUCAA-3′, 5′-UGUGU-3′ and tetrad-forming poly (G) stretches, and avoidance of their presence in the sensitized siRNA, helps overcome Toll-like receptor recognition.

As per Homology-based off-targets, it is very common for siRNA to have multi-targets due to their relatively small length. In fact, both sense and antisense are known to have an off-target effect with several mRNA transcripts [30], [33]. This type of off-target could be subclassified into two subtypes. First type is “Complete or near complete off-target” (Complete homology off-target). Whenever the designed siRNA is completely identical with a region (or with one mismatch) in an unintended mRNA it could lead to the destruction of that mRNA with the same mechanism that siRNA silences the intended mRNA. Alternatively, siRNA could cause “partial off-target” (seed matching off-target) effects, in cases where the designed siRNA seeding region (second to seventh position) matches with 3′UTR of off-target, affecting its translation [34]. This homology based off-target could affect siRNA potency as they become unavailable to bind with the intended mRNA. Therefore, siRNA having off-target effects may be considered undesirable [31], [11]. Several studies have examined the use of chemical modifications to mask siRNA off-target effects, as summarized in [35], [27].

Here, we introduce an automated tool capable of designing siRNA which takes into account multiple transcripts filtration, target accessibility and off-target filtration evaluation in a desktop application named *MysiRNA-Designer*. This is combined with a unique multiple score filtration and efficiency prediction using our specially designed filtration layer. We subsequently applied our strict filtration step on whole human mRNA to demonstrate the practical usage of the tool against experiment datasets and human mRNA.

## Discussion

### Design and Implementation

**Figure 1:**
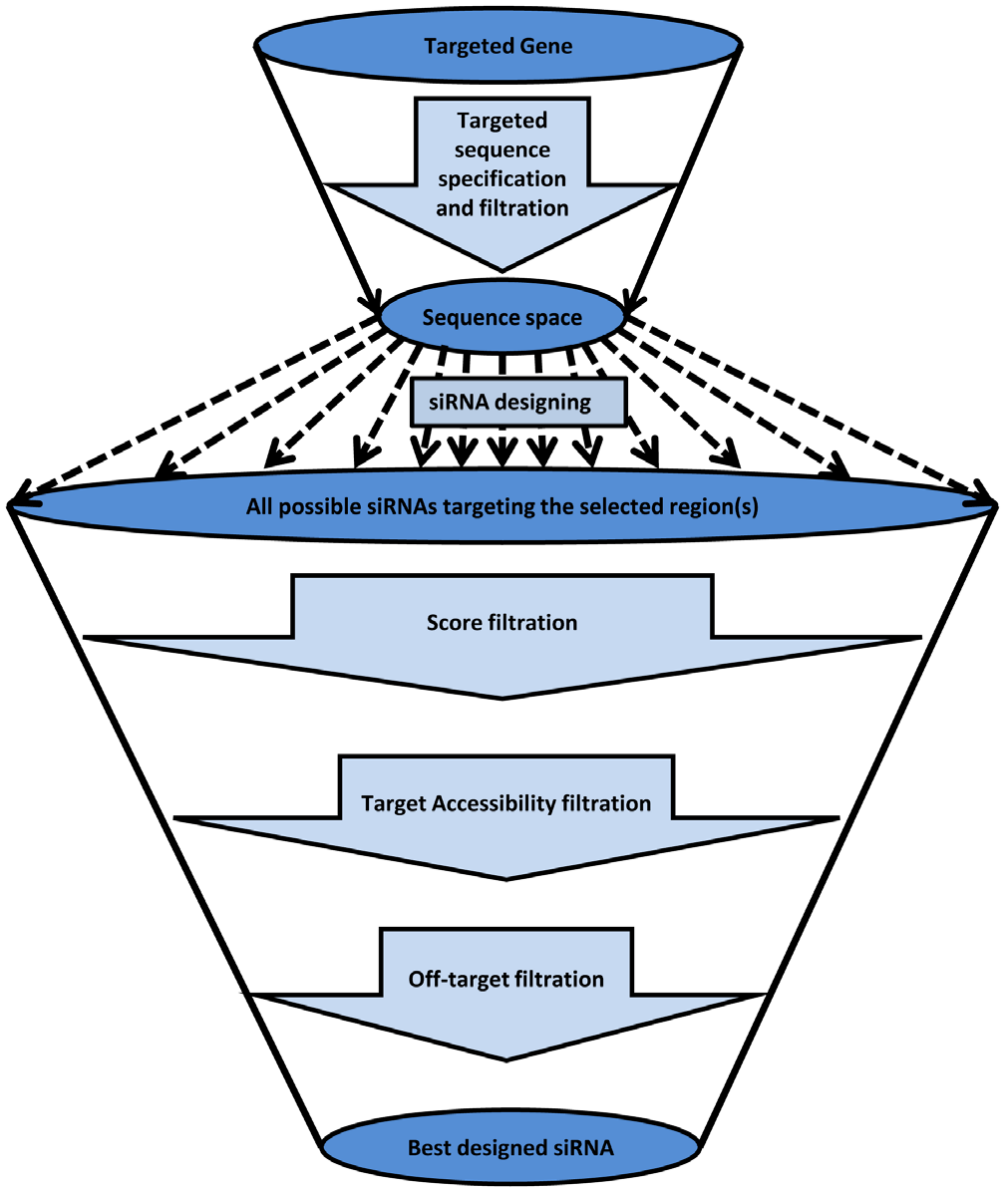
Different phases for designing siRNA with high efficiency & sensitivity. There are seven distinguished phases for siRNA design: 1^st^ choosing the targeted gene for silencing. 2^nd^ identifying the proper target sequence space that represent all gene's transcripts and doesn't have any SNPs. 3^rd^ designing all possible siRNA with nineteen nucleotides length with both sense and antisense strand. 4^th^ these potential siRNAs are scored and evaluated according to several scoring mechanisms and criteria and then filter them according to produced scores. 5^th^ siRNA are filtered according to target accessibility. 6^th^ off-target filtration of the remaining siRNA is performed excluding siRNAs with unwanted off-target effect. 7^th^ select the best designed siRNAs that passes all the previous filtration phases and achieve the highest predicted efficiency.

*MysiRNA-Designer*, presents an automated workflow for siRNA design that implement various scores and state of the art algorithms [**Figure 1**]. It passes through seven phases and filtration steps in order to design double stranded (ds) siRNA with high potential to induce the desired silencing effect. First, the desired gene is targeted via selection of one of its transcripts. Next, sequence space is assigned by examining the targeted mRNA sequence, selection regions that are conserved among the mRNA other transcripts (if any). It is essential to ensure they are free from any single nucleotide polymorphisms (SNPs). Thirdly, all possible siRNAs are designed with the length of 19 nt via one nucleotide shift through the sequence space selected earlier. All these are then subjected to an evaluation step that predict their efficiency using ten state of the art models (see below) as the fourth filtration step. A cut-off score is determined for each of these tools to accept or reject the siRNAs candidates. *MysiRNA-Designer* takes the intersection between all of these tools to increase the specificity and reduce the number of false positive as much as possible. The fifth step considers the evaluation of target accessibility including both secondary structure evaluation and energetic calculation between siRNA strands and siRNA-mRNA. The energetically favoured, target accessible siRNAs pass to the following step where off-target filtration starts, rejecting siRNA that lacks specificity by having off-targeted mRNA(s) either homology based or seed matching based, following a state of the art protocol for off-target evaluation. Finally, all siRNAs that pass these filtration steps are evaluated according to the MysiRNA-model, an artificial neural network model previously described by the authors capable of predicting siRNA efficiency with improved efficiency and sensitivity. We used this model to re-evaluate the siRNA candidates and provide the user with the ability to select the siRNAs passing a specified score level. These steps will be discussed in more detail below.

#### Stage 1–2: Sequence Space Preprocessing

**Figure 2:**
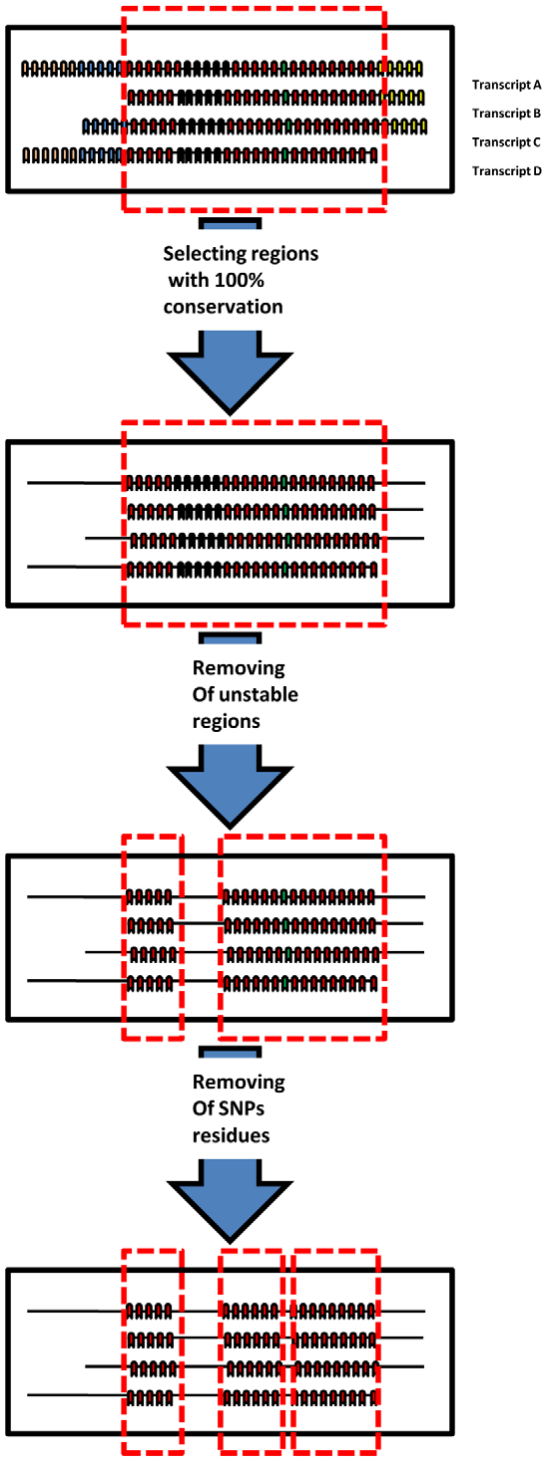
Different preprocessing steps in order to identify the representative sequence space within the mRNA. Sequence space should be free from unstable regions (black color) and SNPs (green color) occurrence, which is conserved among different gene transcripts (red color) which are later, used as a template for siRNA design.

**Figure 3:**
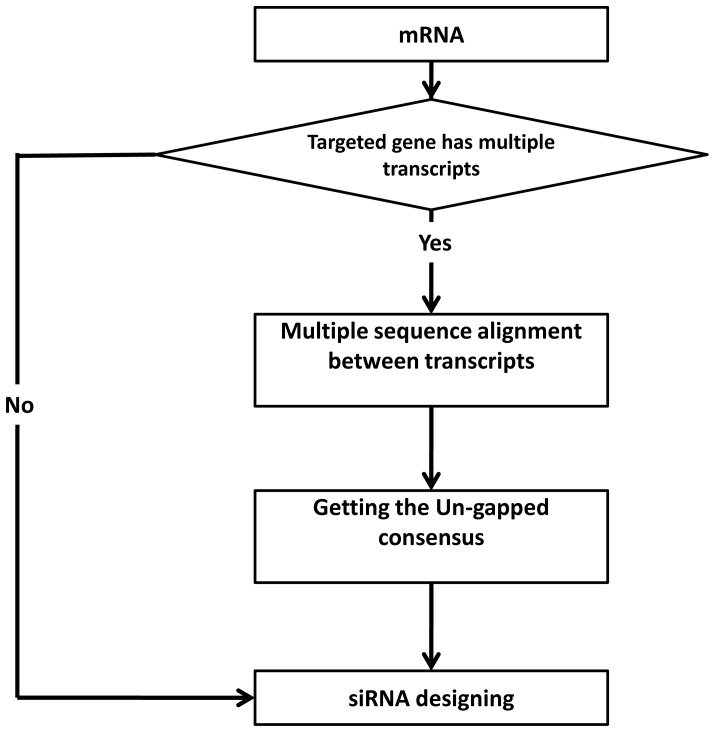
Decision flow of targeted gene's multi-transcript filtration phase implemented in *MysiRNA-Designer*. *MysiRNA-Designer* first check whether the mRNA entered has any other transcripts, if such cases, I get the other transcripts using NCBI blast, and perform multiple sequence alignment to these sequences. The un-gapped consensus is later calculated in order to Design siRNA targeting the desired sequence space.

Various preprocessing techniques were combined to refine the targeted sequence and locate the most representative and conserved region(s) within it. Then, these strict refining constraints were validated. In order to rationally refine the target sequence space, two preprocessing steps were proposed (as a modification of the Birmingham guidelines, [27]) [Figure 2]:

- In case of genes with multiple transcripts, all the gene's transcripts should be targeted to accomplish complete gene silencing. In order to achieve this constraint, all genes' transcripts have to be aligned together and the common regions (conserved regions) located among them. These conserved regions pass to the next step to continue the preprocessing [**Figure 3**].
- The second and final step of preprocessing is the exclusion of single nucleotide polymorphisms (SNPs). SNPs represent small areas (a few nucleotides long) which are known to have high chance of variations (polymorphism). In this step, SNPs residues are excluded, leaving the conserved, stable, SNPs free regions.

After sequence space preprocessing, the proposed multi-score filtration was used to evaluate each potential siRNA and filter them. Only siRNAs that passed this multi-score filtration are considered to be active, so that off-target evaluation could be conducted.

#### Step 3–4: MysiRNA-Designer Multi-Scores Filtration

There are several methods for scoring and predicting the designed siRNA activity, some more accurate than others. However, they are generally classified into two groups (Ichihara et al. 2007): (i) Huesken dataset non dependant [first generation] [9]; and (ii) Huesken dataset dependant [second generation]. The first generation tools depend on differential end GC content evaluation and base pair preferences. These rules have been implemented in models such as Reynolds [2], Amarzguioui [3], Takasaki [4], Katoh [5], Ui-Tei [6] and Hsieh [7]. The second generation tools, on the other hand, developed via extensive examination of the Huesken dataset, comprise models such as Biopredsi [9], DSIR [11], ThermoComposition21 [10] and i-Score [8].

In *MysiRNA-Designer*, a filtration stage is implemented which takes into account high accuracy models, both first and second generation. For the first generation models, the Huesken dataset [9] was examined and active siRNAs were isolated. Then we used these experimentally verified siRNAs to assign threshold scores for each of the first generation models. The data was subjected to a normalization step to remove outliers, using a standard deviation calculation [Table 1]. The second generation tools were handled differently, knowing that these tools aim to predict the siRNA inhibition efficiency (rather than providing scores reflecting rules compliance as in the first generation). A threshold of 70% was assigned for each model, siRNA above this threshold were considered efficient and below this were considered inactive, as per [12]. By taking the intersection between all these models, we developed our multi-scores filtration stage that enables identification of siRNAs capable of producing the silencing efficiency desired. This multi-scores filtration phase reduces the incidence of false positive (i.e. increases the specificity) of the designed siRNA. The efficiency of our proposed multi-scores filtration was demonstrated in a comparative analysis against each of the first and second generation tools, as discussed below.

**Table 1:** Assigned threshhold scores using the Huesken dataset to analyse each scoring tool to two thresholds to filter siRNA with expected inhibition efficiency 90%.

	Min score	Min Threshold	Mean	Max Threshold	Max score	Standard Deviation
**Reynold**	0	1.9	5.52	9.15	10	1.81
**Ui-Tie**	III	III	Ib	Ia	Ia	0.84
**Amarzguioui**	−2	−1.21	2.04	5.3	5	1.62
**Katoh**	31	42.03	69.52	97.01	103.9	13.7
**Hsieh**	−2	−1.11	1	3.11	4	1.05
**Takasaki**	−11	−10.22	1.92	14.06	20.2	6.07

First, siRNA with inhibition efficiency above 90% are isolated from the dataset. Then for each scoring tool, the mean and Standard deviation is calculated and the minimum and maximum thresholds are assigned by deviation from the mean by two folds of standard deviation.

#### Step 5: Target Accessibility

Target accessibility evaluation is a crucial step that affects siRNA inhibition efficiency, as it reflects where the mRNA is more likely to be accessed by short oligomers such as siRNAs. As discussed previously, energetic calculations are required on two occasions, firstly, duplex energy (hybridization energy) and, secondly, opening energy that should be calculated for both siRNA duplex (ds-siRNA) and targeted mRNA. In addition to the total binding energy, RNA secondary structure evaluation should also be taken into account [36]. The siRNA mediated gene silencing is mainly mediated through activation of a complex named an RNA induced silencing complex (RISC) that later binds to the siRNA sequence and to the complementary mRNA [27]. The target accessibility effect on siRNA efficiency is derived from the fact that the RISC is able to bind only to single stranded regions, free from any secondary structures and that the RISC is unable to unfold the RNA structure [37].

Several programs are used to calculate the binding energy, such as *RNAduplex*, *RNAplfold* and *RNAup*, which are capable of calculating the binding energy partially or in total [28]. *RNAduplex*, *RNAplfold* and *RNAup* belong to the Vienna RNA package (available online at http://www.tbi.univie.ac.at/~ivo/RNA/). In *MysiRNA-Designer*, we used the *RNAxs* program in our workflow to evaluate target accessibility. *RNAxs* combines *RNAfold* [38], which predicts RNA secondary structure, with target accessibility energy calculations using *RNAplfold* and *RNAduplex* [26]. *RNAxs* provided two major advantages: a time reduction and a single-phased process. *RNAxs* was included with two other target accessibility based programs (*OligoWalk* & *Sirna*) in a comparative analysis study [36]. Only *RNAxs* was able to identify siRNAs with inhibition efficiency greater than 50%, and classify up to 50% of experimental siRNA. Hence, in *MysiRNA-Designer*, only siRNAs with acceptable target accessibility profile according to *RNAxs* are considered as successful candidates and subjected to further analysis. The detailed *RNAxs* parameters can be found in the supplementary data (Table S1).

#### Step 6: Off Target Filtration

**Figure 4:**
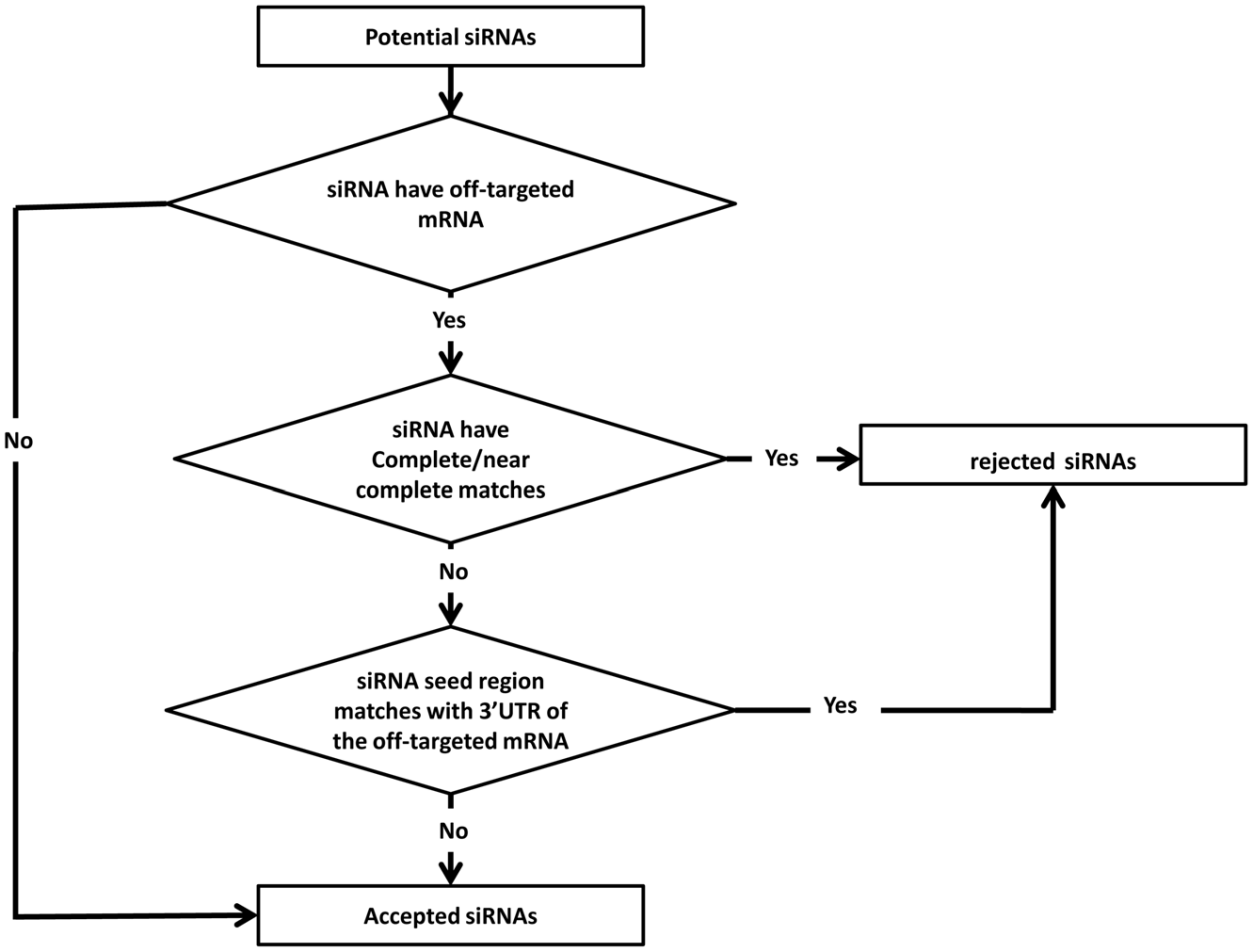
Off-target filtration workflow describing decision making process for siRNAs off-target filtration. Initially, *MysiRNA* checks the existence of off-target for each siRNA, using mRNA reference sequences. In case where off-target has been found, it check whether it is a complete homology (with one or two mismatch), where it is be rejected. In cases free from complete homology, it s check the existence of seed Homology, where the siRNA seed region (2^nd^ to 3^rd^ nts) matches with the off-targeted mRNA 3′UTR. If the siRNA free from both complete homology and seed matching homology it is considered as off-target free and hence pass this filtration step.

The designed siRNA are filtered to evaluate their tendency to trigger off-targets effect using the mRNA dataset. This process is considered the rate-limiting step as it is time consuming to search and evaluate the siRNAs candidates. As discussed previously, there are two types of homology based off-targets, either complete/near-complete off-targets (‘complete homology’) or partial off-targets (‘seed matching’). First, the candidate siRNA is blasted against a mRNA refseq dataset that can be downloaded from (ftp://ftp.ncbi.nih.gov/blast/db/), using *Blastall* to identity complete homology off-targets. siRNAs having 19 (complete) or 18 (near complete) complementarily with the off-targeted mRNA are considered off-targets and are rejected. siRNAs that successfully pass this filtration stage are subjected to another stage to identify siRNA with partial off-targeting tendency seed matching. siRNA that binds using its seed region (2nd to 7th nucleotides from its 3′UTR end) to the off-targeted mRNA is subjected to this stage of evaluation [Figure 4]. The mRNA 3′UTR is downloadable from ensemble at http://www.ensembl.org/index.html. As the default parameters are improper for siRNA blasting, it is very important to adjust blast search parameters as recommended in the work of [27], see supplementary data for detailed Blastn parameters (Table S1). Only siRNAs with no complete homology or seed matching homology with mRNA are accepted.

#### Step 7: Selection of the Best siRNA candidates

The successful candidates from all of the previous steps are finally re-evaluated using the *MysiRNA* model. It acts as second layer neural network combining the whole stacking energy together with two best performing algorithms, enhancing both specificity and sensitivity when compared to other models (as described elsewhere). siRNAs exceeding a score of 93 were considered active. This strict filtration step was able to boost the specificity of the program without significantly affecting the sensitivity (see Results).

### *MysiRNA-Designer* Input, Options, Output, Data and Tools

**Figure 5:**
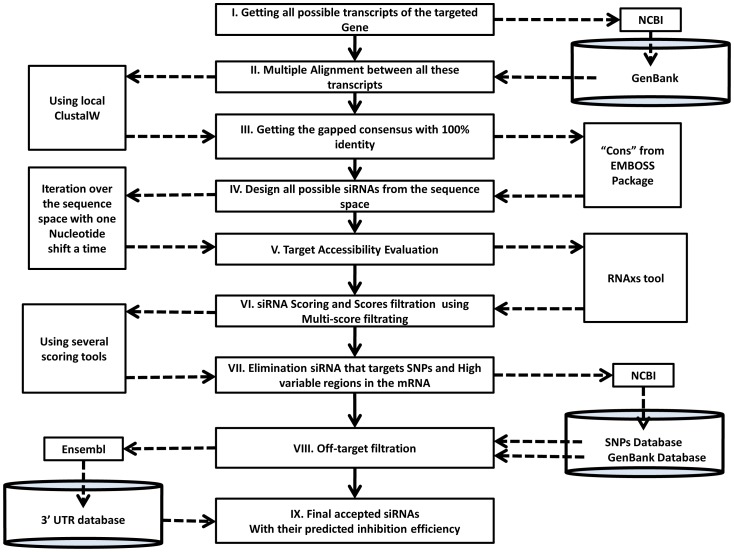
Flow chart of “*MysiRNA-Designer*” program. *MysiRNA-Designer* takes an accession; get the mRNA sequence from NCBI-GenBank. Finds out if this mRNA has other transcript(s), performs multiple sequence alignment with the transcripts, if any, and takes the consensus un-gapped sequence, designs all possible siRNA in targeting sequence space available; performs target accessibility evaluation selection siRNA with energetically and structurally favored siRNA-mRNA binding. Predict siRNA efficiency using the implemented multi-score filtration; select the candidates that pass the threshold assigned for each of the ten tools used, eliminates siRNA targeting SNPs regions or off-targeted mRNA, either complete or seed homology off-target. Finally, *MysiRNA-Designer* shows the accepted candidates with the predicted silencing efficiency using MysiRNA-Model, it filters candidates above the assigned threshold, as the user requires.

By entering the Accession Number (RefSeq-ID assigned for each mRNA), *MysiRNA-Designer* connects to the National Center for Biotechnology Information (http://www.ncbi.nlm.nih.gov/) and obtains the sequence information of that accession number. The software accepts either accession or a list of accessions and offers the user the capability of selecting the *MysiRNA-model* high specificity threshold. Then it finds all available transcripts using remote BLAST (Bioperl package). It performs multiple sequence alignment (MSA) between those transcripts using ClustalW (available at ftp://ftp.ebi.ac.uk/pub/software/clustalw2/). Then, it uses *CONS* tool to get the 100% consensus between these different transcripts (*CONS* belongs to the EMBOSS package at http://www.interactive-biosoftware.com/embosswin/embosswin.html). The software designs of all possible siRNAs within the consensus, using one nucleotide frame shift. These siRNAs are filtered, removing those with any occurrence of Single Nucleotide Polymorphisms (SNPs), then it performs target accessibility evaluation using *RNAxs* [26] combining *RNAfold*, *RNAduplex* and *RNAalifold*. After performing both SNPs filtration and target accessibility filtration, the accepted siRNAs are subjected to our designed multi-score filtration using ten different tools, [2]–[11]. Those siRNA having acceptable scores are subjected to off-target filtration step, as described in Methods [Figure 5]. The output is produced in a ‘fasta-like’ format with various scores of each tool in the header and siRNA antisense and sense sequence in the fasta-body, together with the *MysiRNA*-model score.

## Results

### Comparison and Evaluation of *MysiRNA-Designer* scores to other Algorithms

In this work, a multi-score filtration algorithm was implemented in *MysiRNA-Designer* that takes into account the intersection between ten siRNA scoring tools. These tools were developed using either position preferences rules or a sophisticated data mining approached to evaluate siRNA-mRNA binding and predict the silencing efficiency. Two comparative studies were conducted using the data presented by Fellmann et al [39], consisting of nine genes, in which each possible siRNAs were designed and experimentally tested. First, we compared the specificity of our proposed multi-score filtration technique against the other models. It was found able to separate active siRNAs from inactive ones with enhanced efficiency and specificity, with the least number of false positive siRNA (Specificity of 93%). In the second study, we applied another score filtration layer, which depends on our previously developed model for siRNA efficiency prediction ([40], submitted). This modification was able to enhance the specificity up to 97%, without significantly affecting the sensitivity [Table 2], for detailed results, see Supplementary Data (Table S2).

**Table 2:** Evaluation of the specificity and sensitivity of different models compared to *MysiRNA-Designer* two modes (Intersections of different scoring modelsand MysiRNA model 93% on the Fellmann experimental dataset.

	Ui-Tei	Amar	Hsieh	Taka	Biopredsi	i-Score	Rey	Katoh	DSIR	Thermo21	Multi-Scores	MysiRNA-Model 93%
**Sensitivity**	0.99	0.97	1.00	1.00	0.73	0.32	1.00	0.68	0.85	0.84	0.30	0.22
**Specificity**	0.13	0.13	0.01	0.01	0.69	0.92	0.01	0.68	0.54	0.55	0.93	0.97
**TP**	236	232	238	237	173	72	119	161	203	199	69	24
**FN**	2	6	0	1	65	166	119	77	35	39	169	214
**TN**	2476	2421	268	218	12605	17203	15213	12506	9955	10008	17315	17820
**FP**	15879	15934	18087	18137	5750	1152	3142	5849	8400	8347	1040	535

The combination of multiple scoring tools rather than single one, in our designed multi-scores filtration stage perform with enhanced efficiency when compared against experimental data results [39]. This study involved tools such as: Ui-Tei [6], Amarzguioui [3], Hsieh [7], Takasaki [4], Biopredsi [9], i-Score [8], Reynolds [2], Katoh [5], DSIR [11] and ThermoComposition21 [10], in order as shown in the table. As our aim to reject as much false positive (FP) as possible, the intersection between tools provided solid, more reliable results with specificity up to 93%. In addition, we used MysiRNA-Model, an Artificial Neural Network model for siRNA scoring and efficiency prediction, via assigning a threshold of 93% above it siRNA candidates were considered accepted. This modification was integrated with our multi-score filtration algorithm and was able to boost the specificity up to 97% [**see supplementary data**].

**TP** = true positives, **FN** = false negatives, **TN** = true negatives, **FP** = false negatives.

### Prediction of Active siRNA with *MysiRNA-Designer* on 10% of Human Genome

To test the practicality of this proposed multi-score filtration, 10% of human mRNAs were subjected to this filtration stage within this study. To guarantee the selection of a representative sample, one percent were selected from the mRNA dataset (NCBI mRNA refseq latest release with 46,395 mRNA records) in every 10% (i.e. 464 records were randomly selected every 4639 records), see Supplementary Data (Table S3). This sample covered genes with different transcripts ranging from one to 13 transcripts. In this study, two filtration systems were applied: firstly, multi-score filtrating using the upper and lower threshold for each score [Table 1] and secondly, targeted sequence space limitation only to the consensus between multiple transcripts. After examining the results, it was found that 95.65% of the mRNAs had at least one siRNA that met the selection criteria. This showed the practical usage of this multi-score filtration together with multi-transcript filtration. To study the results of multi-score filtration alone, only genes with single transcript were examined (to eliminate the effect of multiple-transcript filtration). It was found that 96.76% of those mRNAs had at least one siRNA passing this strict filtration condition with an average of 132 siRNA per gene.

### Comparison Between *MysiRNA-Designer* and Several Programs According to the Workflow Implemented

We conducted a comparison between *MysiRNA-Designer* and several siRNA designing tools such as *siDESIGN Center*, *Asi-Designer*, and *RNAxs*. The comparison included stages implemented in each tool, such as the tool's ability to consider multiple transcripts and select conserved region analysis, Target accessibility evaluation, SNPs, and Off-target filtration covering both complete (full homology) and partial (seed region) filtration [Table 3]. These steps represent state of the art approaches for efficient design of siRNAs [41]. As illustrated, *MysiRNA-Designer* performs all of the above mentioned steps required for efficient siRNA design, while the other tools do lack some of the mentioned criteria.

**Table 3:** Comparison between *MysiRNA-Designer* and several programs used for siRNA full automation designing.

*Tools name*	*Multi-transcripts Consideration*	*Conserved Region Analysis*	*SNPs Evaluation*	*Multi- algorithms Scoring*	*2ry structure Evaluation*	*Target accessibility*	*Full Homology Off-target*	*Seed Region off-target*	*Server Based*
***MysiRNA-Designer***	***+***	***+***	***+***	***+***	***+***	***+***	***+***	***+***	***−***
***siDESIGN Center *** *1	***+***	***+***	***+***	***−***	***−***	***−***	***+***	***+***	***+***
***Asi-Designer *** *2	***+***	***−***	***+***	***−***	***+***	***−***	***+***	***−***	***+***
***RNAxs *** *3	***−***	***−***	***−***	***−***	***+***	***+***	***−***	***−***	***+***

This Comparison involves tools ability to perform alignment between different transcripts, conserved regions consideration. All together with siRNA candidate evaluation using several algorithms and target accessibility. siRNAs iltration by the presence of Single Nucleotide Polymorphisms and off-targets (both full homology and seed regions).

*1  ***siDESIGN Center available at*** http://www.dharmacon.com/designcenter/DesignCenterPage.aspx.

*2  ***Asi-Designer available at*** http://sysbio.kribb.re.kr:8080/AsiDesigner/menuDesigner.jsf.

*3  ***RNAxs available at*** http://rna.tbi.univie.ac.at/cgi-bin/RNAxs.

### Comparison between *MysiRNA-Designer* and Several siRNA Design Programs

*MysiRNA-Designer* was involved in a comparative study against other siRNA design tools to assess their ability to select active siRNAs and reject inactive ones. Essentially, these tools are expected to reject as many false positives as possible, while retaining the ability to design one or more active siRNAs. We used the complete data from nine genes, for which each of the possible siRNA was designed and tested [39].

The data shows Short Hairpin RNAs that are processed to siRNAs, and their experimentally verified inhibition efficiency. Three other siRNA design programs were compared to *MysiRNA-Designer* in this study: *siDESIGN Center*, *Asi-Designer* and *RNAxs*. The results of each program were compared to the experimental data results and the results can be subclassified into four types: True Positive (TP) and True Negative (TN) when the program successfully managed to identify active siRNA and inactive siRNA, and False Positive (FP) and False Negative (FN) in cases when the program falsely identified inactive siRNA as active, or active siRNA as inactive, respectively. Both the sensitivity (which reflects the ability to identify true positives) and specificity (which reflects the ability to reject false positives) were taken into consideration. *MysiRNA-Designer* was found capable of designing siRNA with high level of specificity and sensitivity. It achieved a specificity of 0.96 to 0.98 (−/+ MysiRNA-Model score filtration) compared to *AsiDesigner*, *siDesign* and *RNAxs* which achieved 0.95, 0.94 and 0.76, respectively [Table 4], for detailed result see supplementary data (Table S4).

**Table 4:** Illustration of the Comparative analysis results between *MysiRNA-Designer*, AsiDesigner, siDesign and RNAxs against an experimentally verified dataset.

	*Asi-Designer*	*siDesign*	*RNAxs*	*MysiRNA*	*MysiRNA 93%*
***sensitivity***	0.13	0.18	0.50	0.19	0.14
***Specificity***	0.95	0.94	0.76	0.96	0.98
***TP***	31	42	117	44	33
***FN***	201	190	115	188	199
***TN***	17657	17409	14068	17843	18090
***FP***	813	1061	4402	627	380

Using the experimentally verified dataset, published in [39], a comparative analysis involving *MysiRNA-Designer* and three of the top siRNA design programs, that preform whole automation process. We used both *MysiRNA-Designer* options either with or without the implementation of MysiRNA-Model threshold. The result of this study demonistrate the superiority of *MysiRNA-Designer*, in either options, in rejecting as much false positive as possible, reflecting the high spicificity desired.

**TP** = true positives, **FN** = false negatives, **TN** = true negatives, **FP** = false negatives.

It can be interpreted that the inclusion of target accessibility evaluation enhanced the specificity from 0.93 (based on multi-score filtration solely, shown above) to 0.96. These findings demonstrate the superiority in terms of specificity, of *MysiRNA-Designer* over the other tools involved, as 98% of siRNA designed are expected to be active. In addition, they demonstrate the ability of *MysiRNA-Designer* to design both siRNAs and shRNAs. However, the results show a decrease in sensitivity, which may be tolerated, as the main purpose is to reject false positives.

### Conclusion

*MysiRNA-Designer* is free desktop-based software capable of designing siRNA with a high level of specificity and sensitivity. It runs on Microsoft Windows environment, allowing it to be used by the vast majority of users, especially the non-computer experienced scientists. It combines the implementation of several algorithms and state of the art tools for proper siRNA designing. Sequence space is preprocessed, considering differential splicing, to allocate the targeted regions. Several filtration steps take place as SNPs filtration, target accessibility filtration, multi-score filtration and off-target filtration. *MysiRNA-Designer* was tested against human mRNA and experimental data and achieved improvements in the results obtained by other similar tools. Hence, we believe it may play a key role in this field. *MysiRNA-Designer* is a freely accessible through the journal supplementary data, MysiRNA-Designer S1. For information about the installation instructions, installation validation results and source code please refer to Readme S1, Testing S1 and Source S1, respectively.

## Supporting Information

Table S1 **Blast and RNAxs running parameters.** RNAxs parameters capable of performing target accessibility evaluation for siRNA-mRNA shall be modified as per above, [36]. The default BLASTn running parameters are inappropriate to performing siRNA off-target dataset search due to their small length, therefore word size, expect value, mismatch, gap opening and gap extension penalty shall be modified as illustrated, [27].
(PDF)

Table S2 **Comparison and Evaluation of** ***MysiRNA-Designer*** **scores to other Algorithms.** Two comparative studies were conducted using the data presented by Fellmann et al [39], between ur proposed multi-score filtration phase and ten other tools: Reynolds [2], Amarzguioui [3], Takasaki [4], Katoh [5], Ui-Tei [6], Hsieh [7], Biopredsi [9], DSIR [11], ThermoComposition21 [10] and i-Score [8]. First, we compared the specificity of our proposed multi-score filtration technique against the other models. It was found able to achieve specificity of 93%. In the second study, we applied another score filtration layer, which depends on our previously developed model for siRNA efficiency prediction ([40], submitted) enhancing the specificity up to 97%.
(XLSX)

Table S3 **Ten percent of the Human RefSeq Genes used to evaluate** ***MysiRNA-Designer*** **.** One percent were selected from the mRNA dataset (NCBI mRNA refseq latest release with 46,395 mRNA records) in every 10% (i.e. 464 records were randomly selected every 4639 records)., to guarantee the selection of a representative sample.
(XLSX)

Table S4 **Detailed illustration of the Comparative analysis results between** ***MysiRNA-Designer*** **, AsiDesigner, siDesign and RNAxs against an experimentally verified dataset.** Using complete data of nine genes, where which each of the possible siRNA was designed and tested [39], to compare *MysiRNA-Designer* was compared to *siDESIGN Center*, *Asi-Designer* and *RNAx*. The specificity and sensitivity of each tool were calculated, indicating the improvement achieved b*y MysiRNA-Designer*.
(PDF)

Readme S1 **MysiRNA-Designer installation instruction.**
(TXT)

Testing S1 **MysiRNA-Designer installation validation results.** Illustration of siRNA targeting mRNA (acc: NM_001667) using MysiRNA-Designer. It presents 22 possible siRNA with their predicted efficiency.
(TXT)

Source S1 **MysiRNA-Designer source code.**
(PL) Click here for additional data file.
