# Correction: MysiRNA-Designer: A Workflow for Efficient siRNA Design

**DOI:** 10.1371/journal.pone.0119062

**Published:** 2015-03-18

**Authors:** The PLOS ONE Staff

## Notice of Republication

This article was republished on January 27, 2015, to remove Supporting Information file MysiRNA-Designer S1, which was infected. The publisher’s attempts to contact the author for a replacement file were unsuccessful. Please download this article again to view the updated version.

## References

[1] Mysara M, Garibaldi JM, ElHefnawi M (2011) MysiRNA-Designer: A Workflow for Efficient siRNA Design. PLoS ONE 6(10): e25642 doi:10.1371/journal.pone.0025642 22046244 10.1371/journal.pone.0025642 PMC3202522

