# Supplementary material for: MysiRNA-Designer: A Workflow for Efficient siRNA Design
Source: PLoS One. 2011 Oct 26;6(10):e25642. doi: 10.1371/journal.pone.0025642 (PMC3202522; doi:10.1371/journal.pone.0025642)
Supplement: Table S1 — Blast and RNAxs running parameters. RNAxs parameters capable of performing target accessibility evaluation for siRNA-mRNA shall be modified as per above, [36]. The default BLASTn running parameters are inappropriate to performing siRNA off-target dataset search due to their small length, therefore word size, expect value, mismatch, gap opening and gap extension penalty shall be modified as illustrated, [27]. (PDF) [file pone.0025642.s001.pdf]

Blast and RNAxs running parameters.

RNAxs parameters for siRNA		***Blast parameters for siRNA***	
Parameter	***Adjustment***	***Parameter***	***Adjustment***
Window size	*80*	***Word size***	*11*
16-nt accessibility threshold	*0.001002*	***Expect threshold***	*10*
8-nt accessibility threshold	*0.01157*	***Mismatch penalty***	*3*
Maximum base-pair distance	*140*	***Gap open penalty***	*5*
Gap extension penalty	*2*	***Gap extension penalty***	*2*

The default blast running parameters are inappropriate to performing siRNA off-target dataset search due to their small length, therefore they needed to be modified. RNAxs parameters capable of performing target accessibility evaluation for siRNA-mRNA.
