# Supplementary material for: MysiRNA-Designer: A Workflow for Efficient siRNA Design
Source: PLoS One. 2011 Oct 26;6(10):e25642. doi: 10.1371/journal.pone.0025642 (PMC3202522; doi:10.1371/journal.pone.0025642)
Supplement: Table S4 — Detailed illustration of the Comparative analysis results between MysiRNA-Designer , AsiDesigner, siDesign and RNAxs against an experimentally verified dataset. Using complete data of nine genes, where which each of the possible siRNA was designed and tested [39], to compare MysiRNA-Designer was compared to siDESIGN Center, Asi-Designer and RNAx. The specificity and sensitivity of each tool were calculated, indicating the improvement achieved by MysiRNA-Designer. (PDF) [file pone.0025642.s004.pdf]

Datied illustration of the Comparative analysis results between *MysiRNA-Designer*, AsiDesigner, siDesign and RNAxs against an experimentally verified dataset.

	Asi-designer	siDesign	RNAxs	MysiRNA	MysiRNA 93%		Asi-designer	siDesign	RNAxs	MysiRNA	MysiRNA 93%
			**Bcl2**						**MYC**		
TP	3	4	8	3	3	TP	3	3	12	2	1
FN	16	15	11	16	16	FN	19	19	10	20	21
TN	1852	1839	1571	1870	1885	TN	2248	2232	1856	2272	2299
FP	66	79	347	48	33	FP	80	96	472	56	29
Sensitivity	0.16	0.21	0.42	0.16	0.16	Sensitivity	0.14	0.14	0.55	0.09	0.05
Specificity	0.97	0.96	0.82	0.97	0.98	Specificity	0.97	0.96	0.80	0.98	0.99
			**Hras1**						**Pcna**		
TP	2	0	3	1	1	TP	3	7	13	8	6
FN	3	5	2	4	4	FN	20	16	10	15	17
TN	491	466	426	506	512	TN	1111	1116	897	1142	1154
FP	25	50	90	10	4	FP	77	72	291	46	34
Sensitivity	0.40	0.00	0.60	0.20	0.20	Sensitivity	0.13	0.30	0.57	0.35	0.26
Specificity	0.95	0.90	0.83	0.98	0.99	Specificity	0.94	0.94	0.76	0.96	0.97
			**Kras**						**Rpa3**		
TP	9	11	37	16	13	TP	0	1	2	0	0
FN	83	81	55	76	79	FN	3	2	1	3	3
TN	4306	4264	2975	4293	4383	TN	531	520	379	521	534
FP	215	257	1546	228	138	FP	26	37	178	36	23
Sensitivity	0.10	0.12	0.40	0.17	0.14	Sensitivity	0.00	0.33	0.67	0.00	0.00
Specificity	0.95	0.94	0.66	0.95	0.97	Specificity	0.95	0.93	0.68	0.94	0.96
			**Mcl1**						**Trp53**		
TP	6	5	29	12	7	TP	1	1	3	1	1
FN	40	41	17	34	39	FN	3	3	1	3	3
TN	3246	3219	2661	3287	3328	TN	1661	1679	1422	1697	1719
FP	157	184	742	116	75	FP	68	50	307	32	10
Sensitivity	0.13	0.11	0.63	0.26	0.15	Sensitivity	0.25	0.25	0.75	0.25	0.25
Specificity	0.95	0.95	0.78	0.97	0.98	Specificity	0.96	0.97	0.82	0.98	0.99
			**Hmyc**						**Total**		
TP	4	10	10	1	1	TP	31	42	117	44	33
FN	14	8	8	17	17	FN	201	190	115	188	199
TN	2211	2074	1881	2255	2276	TN	17657	17409	14068	17843	18090
FP	99	236	429	55	34	FP	813	1061	4402	627	380
Sensitivity	0.22	0.56	0.56	0.06	0.06	Sensitivity	0.13	0.18	0.50	0.19	0.14
Specificity	0.96	0.90	0.81	0.98	0.99	Specificity	0.95	0.94	0.76	0.97	0.98
